# Efficacy and safety of edaravone dexborneol in acute ischemic stroke: systematic review and meta-analysis

**DOI:** 10.3389/fneur.2025.1649476

**Published:** 2025-10-03

**Authors:** Khaled Moghib, Mahmoud Tarek Hefnawy, Shehab M. Moawad, Izere Salomon, Ahmed Hamdi, Olivier Uwishema, Mostafa Meshref

**Affiliations:** ^1^Faculty of Medicine, Kasr Al-Ainy Cairo University, Cairo, Egypt; ^2^Medical Research Group of Egypt, Negida Academy, Arlington, MA, United States; ^3^Faculty of Medicine, Zagazig University, Zagazig, Egypt; ^4^University of Rwanda College of Medicine and Health Sciences, Kigali, Rwanda; ^5^Department of Research and Education, Oli Health Magazine Organization, Kigali, Rwanda; ^6^Department of Neurology, Faculty of Medicine, Al-Azhar University, Cairo, Egypt

**Keywords:** edaravone dexborneol, efficacy and safety, stroke, acute ischemic stroke, NIHSS, systematic review

## Abstract

**Background:**

Edaravone dexborneol represents a novel neuroprotective agent utilized in the treatment of acute ischemic stroke (AIS). Preliminary studies indicate that this combination exhibits enhanced therapeutic effects when compared to the use of edaravone alone. The objective of this study was to assess the efficacy and safety of edaravone dexborneol in the management of AIS.

**Method:**

This systematic review and meta-analysis were conducted following the Preferred Reporting Items for Systematic Reviews and Meta-analyses (PRISMA) statement guidelines. A comprehensive search of the PubMed, Cochrane Central, Scopus, and Web of Science databases was performed on December 30, 2024. Subsequently, we screened articles for eligibility, relevant data were extracted, and the risk of bias was assessed utilizing the Cochrane Collaboration Tool 2. The primary outcome evaluated was the efficacy of edaravone dexborneol in the management of AIS, as measured by the National Institutes of Health Stroke Scale (NIHSS) and the modified Rankin Scale (mRS). Secondary outcomes encompassed improvements in activities of daily living (ADL), reductions in post-stroke depression, inflammation, and hemorrhagic transformation, as well as enhancements in cognitive function, as indicated by Montreal Cognitive Assessment (MoCA) scores. Extracted data from pertinent Randomized Controlled Trials (RCTs) were analyzed using R programming for Windows. All procedures outlined in this study were pre-specified, and the protocol has been registered with PROSPERO under the unique identifier CRD42024626320.

**Results:**

A total of six randomized controlled trials (RCTs) and one cohort study, all conducted in China and involving 2,942 patients with ischemic stroke (65.6% male), were included. Treatment regimens consisted of intravenous or sublingual edaravone dexborneol administered for 10–14 days. The pooled analysis of functional outcomes at 90 days, based on five studies, demonstrated a significant benefit, with a 39.5% higher likelihood of achieving favorable mRS scores (OR = 1.40, 95% CI: 1.18–1.65, *p* = 0.0001), without evidence of heterogeneity (I^2^ = 0%). In contrast, pooled analysis of NIHSS outcomes across seven studies using a random-effects model was not significant (SMD = −0.113, 95% CI: −0.333 to 0.107, *p* = 0.314), with substantial heterogeneity (*I*^2^ = 72.7%). However, under the common-effect model, a small but statistically significant benefit was observed (SMD = −0.083, 95% CI: −0.159 to −0.008, *p* = 0.030). Sensitivity analyses indicated that several studies (Fu 2024, Hu 2023, Xu 2019, Xu 2024) attenuated the pooled effect, while exclusion of Li 2024 and Hu 2023 reduced heterogeneity to 40.7% but resulted in only borderline significance. Secondary endpoints consistently demonstrated favorable effects, including improved activities of daily living, enhanced cognitive function (MoCA scores), and reduced rates of post-stroke depression, inflammation, and hemorrhagic transformation. Safety analyses revealed that adverse events were generally mild and comparable to controls, with some evidence suggesting a reduction in serious complications such as hemorrhagic transformation.

**Conclusion:**

Edaravone dexborneol exhibits considerable potential as a neuroprotective agent in the context of AIS, providing both functional and cognitive advantages, alongside a favorable safety profile. The promising efficacy of this compound underscores the necessity for further comprehensive global studies aimed at optimizing its application and enhancing its relevance across diverse populations.

**Systematic review registration:**

https://www.crd.york.ac.uk/prospero/, identifier CRD42024626320.

## 1 Introduction

Acute Ischemic Stroke (AIS) constitutes a significant and devastating cause of adult morbidity and mortality globally, with an estimated annual death toll of 5.5 million (1, 2). Despite advancements in medical interventions, recombinant tissue plasminogen activator (rTPA or alteplase) and endovascular thrombectomy remain the predominant and most efficacious therapeutic options. Nevertheless, approximately 50% of AIS cases encounter ineffective reperfusion (3, 4), which severely impacts patients’ quality of life and imposes substantial economic burdens on families and society (3–5). In response to these shortcomings, research efforts are increasingly focused on novel therapies aimed at enhancing affordability and accessibility in the treatment of this disease. Distinct from traditional approaches that primarily target the reopening of occluded vessels, neuroprotective agents adopt a different strategy by safeguarding the brain itself (6, 7). Numerous clinical trials investigating neuroprotective agents for AIS have not demonstrated significant clinical benefits. For instance, the SAINT I and II trials revealed that the neuroprotective agent NXY-059 was ineffective in treating AIS within 6 h following stroke onset. In a similar vein, nerinetide did not yield improvements in functional outcomes post-endovascular therapy, and the ALIAS trials indicated that a 25% albumin solution (2 g/kg IV) failed to enhance clinical outcomes at the 90-day mark while concurrently increasing the incidence of pulmonary edema and intracerebral hemorrhage. Furthermore, magnesium sulfate administered within 2 h after stroke onset did not improve functional outcomes at 90 days. These limitations emphasize the critical need for an effective neuroprotective agent capable of reducing disability and mortality rates in the management of AIS (8–12).

The novel neuroprotective agent, edaravone dexborneol, comprises antioxidant and anti-inflammatory components that aim to reduce oxidative stress, prevent cell death, and mitigate the detrimental inflammatory response initiated during and after a stroke (3, 7). This agent has demonstrated promising efficacy in treating AIS in a phase III, randomized, double-blind, parallel, comparative study that included 1,200 AIS participants, resulting in improvements in functional outcomes at 90 days (6). Prior studies have indicated that edaravone dexborneol elicits an inflammatory regulatory response, leading to enhanced blood–brain barrier permeability and the promotion of microglial activation towards the M2 phenotype through the modulation of aryl hydrocarbon receptor (AhR) expression (13, 14).

This study represents the first effort of its kind to systematically review the available evidence regarding the safety and efficacy of edaravone dexborneol in the context of AIS. By synthesizing findings from existing research, this analysis aims to deepen the understanding of this novel therapy’s potential, address existing knowledge gaps, and inform its future application in clinical practice.

## 2 Methodology

This research was conducted following the Cochrane Handbook for Systematic Reviews of Interventions (15). The findings were reported following the guidelines established by the Preferred Reporting Items for Systematic Reviews and Meta-analyses (PRISMA) statement (16). Furthermore, this study was registered with PROSPERO, assigned the identification number CRD42024626320.

### 2.1 Information sources and search strategy

We conducted a comprehensive search of four electronic databases, including PubMed, Cochrane Library, Web of Science, and Scopus, spanning from the inception of these databases until December 30, 2024. The search imposed no restrictions regarding the year, gender, or geographical location of studies; however, it was confined to articles published in English. The keywords utilized in the search encompassed “edaravone-dexborneol” and “acute ischemic stroke (AIS),” employing the following search strategy: (“Edaravone Dexborneol”[Title/Abstract] OR “Edaravone and Dexborneol”[Title/Abstract] OR “dual neuroprotection”[Title/Abstract]) AND (“acute ischemic stroke”[MeSH] OR “ischemic stroke”[Title/Abstract]) AND (“efficacy”[Title/Abstract] OR “safety”[Title/Abstract] OR “outcome”[Title/Abstract]).

### 2.2 Eligibility criteria

We included the studies according to the following inclusion and exclusion criteria:

Inclusion criteria:

- *Population (P):* Patients of any age diagnosed with AIS regardless of the route of drug administration.
- *Intervention (I):* Edaravone dexborneol was administered according to the “Guidelines for the Diagnosis and Treatment of AIS 2018.” The duration of treatment was 14 days (17). Edaravone dexborneol as part of AIS management, with or without additional therapies including routine oxygen therapy, blood pressure control (amlodipine besylate or metoprolol), blood glucose control (metformin or insulin), lipid-lowering therapy (atorvastatin or rosuvastatin), antiplatelet aggregation therapy (aspirin or clopidogrel), and anticoagulation therapy (rivaroxaban).
- *Comparator (C):* Edaravone alone, placebo, or the standard ischemic stroke treatments mentioned above.
- *Outcomes (O):* Clinical outcomes, such as neurological improvement, functional recovery, and safety profile of edaravone dexborneol.
- *Study Design (S):* Randomized controlled trials and observational studies published in English.

Exclusion criteria: Reviews, conference abstracts, and non-English language studies were excluded. There were no other restrictions on the study design or population.

### 2.3 Study selection and data extraction

The eligibility screening process was conducted in two phases: first, the titles and abstracts were screened, followed by the retrieval of full-text articles for those abstracts deemed eligible to undergo further assessment against specified eligibility criteria for the meta-analysis. Any disagreements that arose were addressed through discussion. For data extraction, an online data extraction template was developed based on a pre-specified uniform data extraction sheet, encompassing the following domains: (1) characteristics of the included studies, (2) characteristics of the study population, (3) risk of bias domains, and (4) outcome measures.

### 2.4 Risk of bias and quality assessment

The revised Cochrane risk-of-bias tool (RoB 2) for RCTs was employed to assess the risk of bias in the included clinical trials (18). This thorough evaluation addressed critical domains, including the randomization process, sequence allocation concealment, deviations from intended interventions, and the implementation of suitable statistical methods to evaluate intervention effects. Furthermore, it examined outcome measurement, reporting biases, and the overall risk of bias. Studies were categorized according to their methodological quality as exhibiting low risk, some concerns, or high risk of bias. Any discrepancies that arose were resolved by a final determination made by the senior author. For observational studies, the Newcastle-Ottawa Scale (NOS) was utilized (19). This tool allows researchers to adopt a point-based system to classify studies as ‘good,’ ‘fair,’ or ‘poor.’ Disagreements were addressed through dialogue, and a third author was consulted when necessary to ensure consensus.

### 2.5 Calculation of missing data

Whenever data were presented as medians and interquartile ranges (IQR), they were converted to means (M) and standard deviations (SD) utilizing the equations established by Wan et al. (20). In instances where the SD was not provided, it was derived from the standard error using the formula for a single sample: SD = SE * √n (21), where n represents the sample size. In cases where the mean change (MC) between baseline and endpoint was unavailable, it was computed from the pre-treatment and post-treatment means using the equation MC = Mpost-treatment - Mpre-treatment. Furthermore, if the standard deviation of the mean change was not specified, it was calculated from the pre-treatment and post-treatment standard deviations employing the formula SD_change = √(SD_pre^2^ + SD_post^2^–2*r*SD_pre*SD_post) (22). Missing effect sizes (d) and effect size correlations (r) were determined through the following formulas: d = (Mtreatment - Mcontrol)/SDpooled, where SDpooled = √[(SD^2^treatment + SD^2^control)/2], (23) and r = d/√(d^2^ + 4) (24).

### 2.6 Assessment of heterogeneity

A thorough visual inspection of the final forest plots was conducted, complemented by an assessment utilizing *I*-square and Chi-Square tests, specifically Cochran’s *Q* test, to evaluate the extent of heterogeneity. In instances of significant heterogeneity, defined as Chi-Square *p* < 0.1, a sensitivity analysis was executed to address this heterogeneity. This analysis was performed using R programming version 4.4.1 for Windows (25).

### 2.7 Publication bias

**Figure 1:**
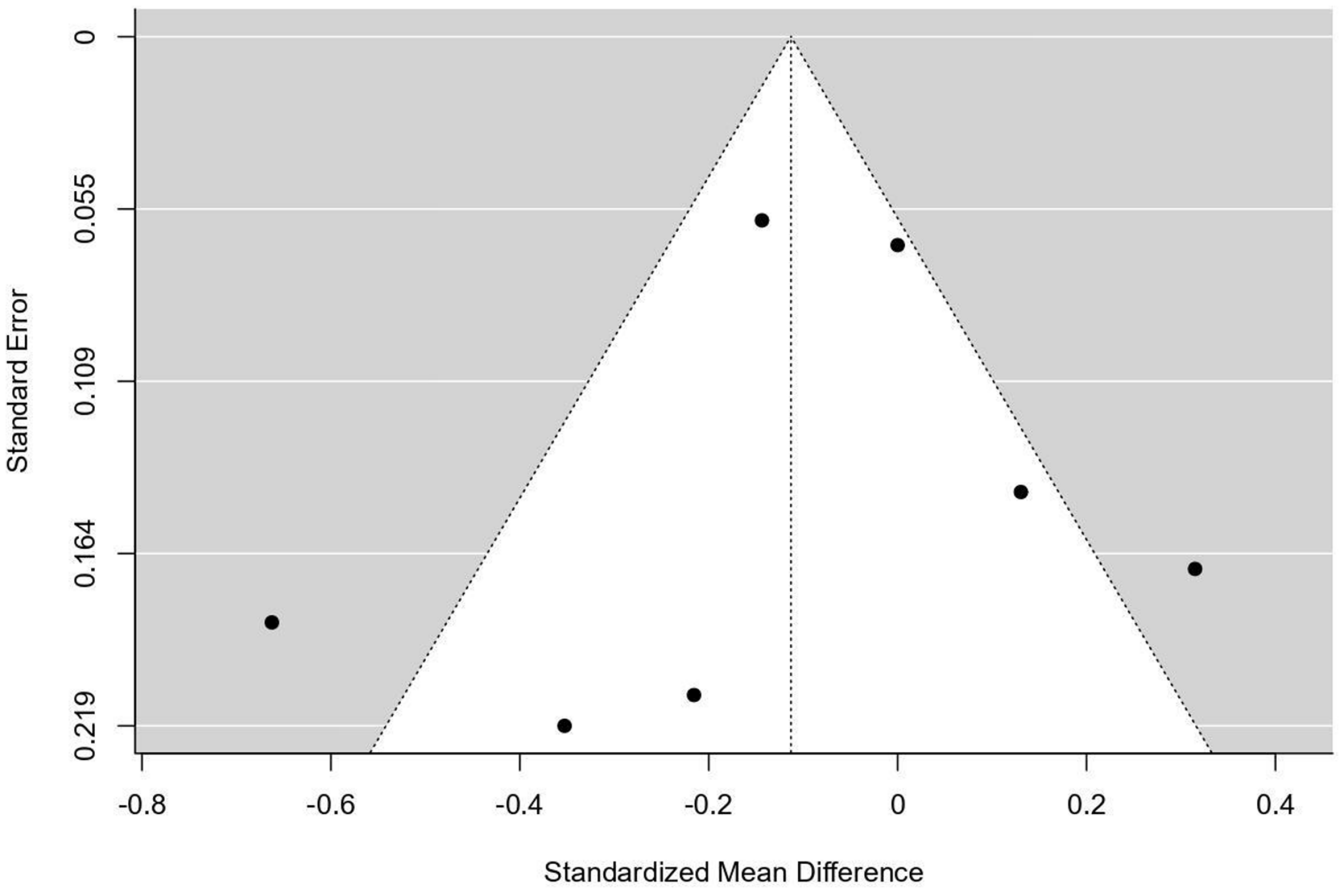
Publication bias, Egger’s test.

A variety of statistical methods were utilized to evaluate publication bias. The Fail-Safe N test, employing the Rosenthal methodology, was conducted to ascertain the number of additional null studies necessary to render the overall meta-analysis results non-significant. Begg and Mazumdar’s rank correlation test was implemented to assess the relationship between effect sizes and their standard errors, thereby identifying potential asymmetry within the funnel plot. Additionally, Egger’s regression test was performed to evaluate the linear relationship between effect size and precision, offering further insight into potential funnel plot asymmetry (Table 1 and Figure 1). Collectively, these methods provide a thorough assessment of potential publication bias in the analysis (26–28).

**Table 1:** Showing publication bias tests.

Publication bias assessment		
Test name	Value	*p*
Fail-Safe N	6.00	0.013
Begg and Mazumdar Rank Correlation (Unreliable)	−0.143	0.773
Egger’s Regression (Unreliable)	−0.730	0.465

Fail-safe N Calculation Using the Rosenthal Approach.

### 2.8 Statistical analysis and choice of the meta-analysis model

The analysis was conducted utilizing the standardized mean difference as the primary outcome measure. A random effects model was employed to fit the data. The degree of heterogeneity, represented as tau^2^, was estimated using the restricted maximum-likelihood estimator (Viechtbauer, 2005). In addition to the estimation of tau^2^, the *Q*-test for heterogeneity (Cochran, 1954) and the *I*^2^ statistic were also reported. Should any degree of heterogeneity be detected (i.e., tau^2^ > 0, independent of the *Q*-test results), a prediction interval for the true outcomes is provided.

To assess potential outliers and influential studies within the model context, studentized residuals and Cook’s distances were analyzed. Studies exhibiting a studentized residual exceeding the 100 × (1–0.05/(2 × k))th percentile of a standard normal distribution were classified as potential outliers, employing a Bonferroni correction with a two-sided alpha level of 0.05 for k studies included in the meta-analysis. Furthermore, studies with a Cook’s distance exceeding the median plus six times the interquartile range of Cook’s distances were deemed influential.

Rank correlation and regression tests were utilized to evaluate funnel plot asymmetry, employing the standard error of the observed outcomes as predictors. All analyses were executed using R Statistical Software (version 4.1.2; R Core Team, 2021), while the risk of bias assessment was conducted using Jamovi version 2.6 for Windows.

### 2.9 Ethical approval

Since this study utilized publicly available data and did not involve direct interaction with human or animal subjects, ethical approval was not necessary. Furthermore, this study was registered with PROSPERO, assigned the identification number CRD42024626320.

## 3 Results

### 3.1 Search and screening

**Figure 2:**
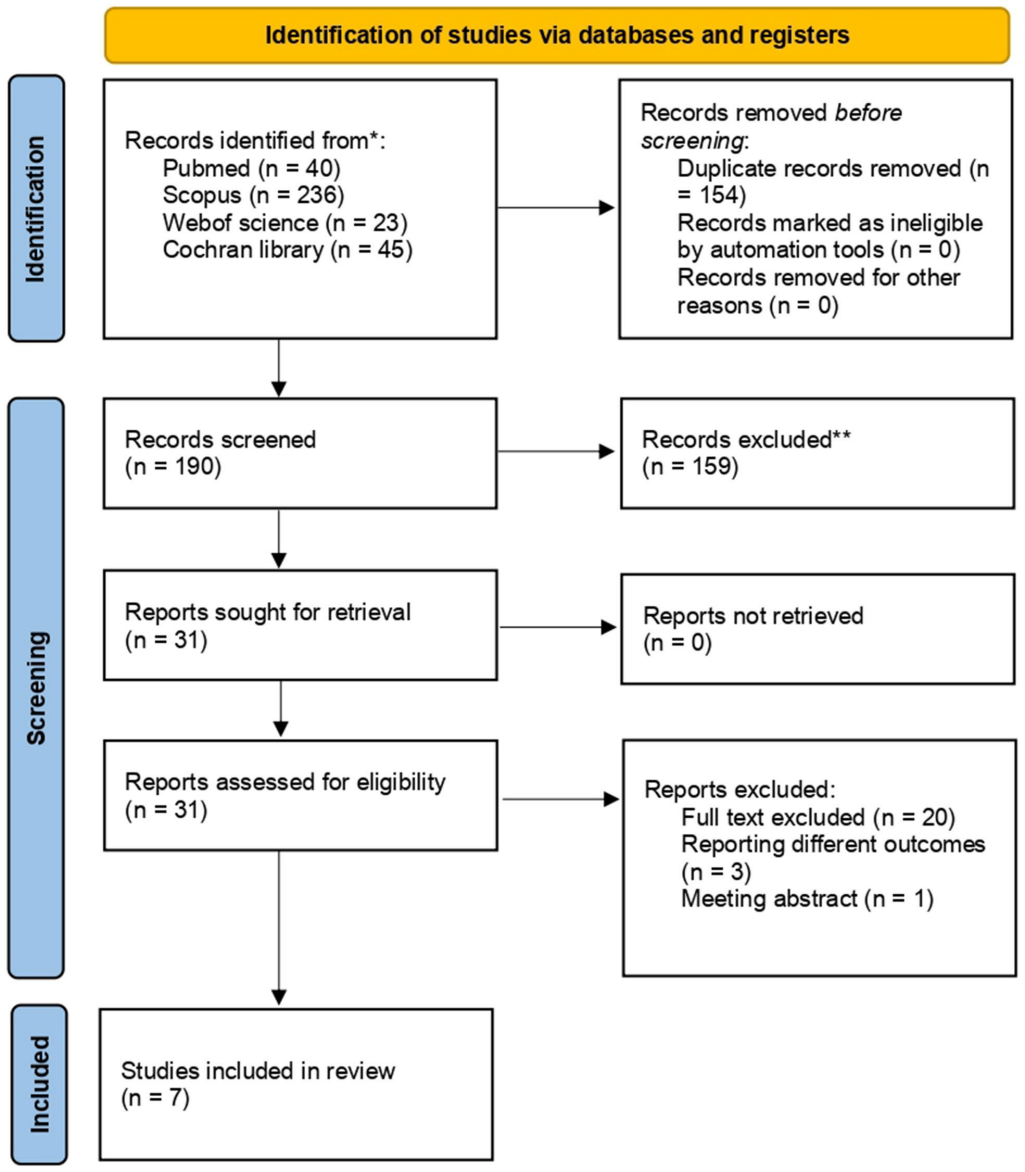
Prisma flow diagram.

Our search initially identified 344 potentially relevant studies. After removing 154 duplicates, 190 records were retained for title and abstract screening. Following this screening, 31 studies were deemed eligible for a full-text review. Of these, six randomized controlled trials (RCTs) and one observational (6, 7, 14, 29–32) encompassing a total of 2,942 patients met the inclusion criteria and were included in the analysis. Twenty publications were excluded during full-text screening: three RCTs did not meet the outcome inclusion criteria, and one publication was only available as an abstract. Further details are provided in Figure 2.

### 3.2 Summary of studies’ characteristics

Of the seven included studies, six were parallel RCTs and one cohort study conducted in China that investigated the effects of Edaravone and Dexborneol in ischemic stroke patients (6, 7, 14, 29–32). Six RCTs and one cohort with 2,942 patients were included, of whom 1931 (65.64%) were males. The treatment regimens involved intravenous and sublingual administration of Edaravone and Dexborneol, either alone or in combination with other standard ischemic stroke treatments, for durations ranging from 10 to 14 days. The participant numbers varied from 85 to 1,200, with a mean age of 60–68. These studies consistently reported significant improvements in functional outcomes, including enhanced modified Rankin Scale (mRS) scores and reduced National Institutes of Health Stroke Scale (NIHSS) scores, indicating better stroke recovery. Xu et al. (29) and Li et al. (5, 30) also observed improved activities of daily living (ADL). In contrast, several studies, such as Hu et al. (31) and Xu et al. (29), noted reductions in post-stroke depression (PSD), inflammation, and hemorrhagic transformation. Improvements in cognitive function, including better MoCA and Stroke Impact Scale scores, have also been highlighted in several trials. Additionally, the studies demonstrated a favorable safety profile, with fewer adverse events compared to standard treatments, as noted by Xu et al. (6).

Notably, the included studies varied in dosing regimens, administration routes, and concomitant therapies. Most trials administered edaravone dexborneol intravenously at 37.5 mg twice daily for 10–14 days, although some explored higher (62.5 mg) or lower (12.5 mg) IV doses (14), and one large RCT (7) evaluated a 36 mg sublingual formulation. Comparator groups also differed, ranging from placebo or standardized ischemic stroke care to active comparators such as edaravone alone (6, 14) and alteplase therapy (5, 30). These variations in regimen and background therapy may partially account for the heterogeneity observed in NIHSS outcomes (Table 2).

**Table 2:** Study characteristics.

Author, year	Country	Study type	Sample size	Age mean (SD)	Male/female	BMI mean (SD)(kg/m^2^)	Blood pressure Systolic/ Diastolic (mm Hg)		Intervention (dose, route, and name)	Comparator (dose, route, and name)	Follow-up (days)	Aim of the study	Findings
Chen et al. (2024) (32)	China	Observational, single-center cohort	85	67.27 (±11.30)	67/18	N/R	N/R	N/R	37.5 mg IV every 12 h (Edaravone Dexborneol) for 14 days	Standardized treatment (antiplatelets/anticoagulants, statins) without Edaravone or Dexborneol	90	Investigated the impact of edaravone dexborneol on functional outcomes and systemic inflammation in AIS patients.	Improved mRS scores (70.7% vs. 47.8% favorable outcome), lower NIHSS scores on days 10–14, and better functional outcome at 90 days.
Fu et al. (2024) (7)	China	RCT (phase III)	914	64.0 (IQR: 56–70)	608/306	Int 24.6 (22.5–26.6)/PL 24.7 (22.5–26.8)	Int 147 (135–162)/PL 147 (136–160)	Int86 (79–95)/ PL86 (80–94)	36 mg sublingual (Edaravone 30 mg, Dexborneol 6 mg), twice daily for 14 days	Placebo (0 mg edaravone, 60 μg dexborneol), sublingual	90	Evaluated the efficacy and safety of sublingual edaravone dexborneol on 90-day functional outcomes in AIS patients.	Improved mRS scores at day 90, reduced NIHSS scores, fewer adverse events, and improved safety outcomes.
Hu et al. (2023) (31)	China	RCT	142	65.8 (±11.0) treatment, 66.6 (±12.7) control	89/53	N/R	N/R	N/R	37.5 mg IV twice daily for 10–14 days (edaravone 30 mg, dexborneol 7.5 mg)	Standardized treatment without Edaravone or Dexborneol	90	Assessed the effects of edaravone dexborneol on neurological function and serum inflammatory factors in acute anterior circulation large vessel occlusion stroke.	Improved mRS scores ≤1, reduced NIHSS scores at 90 days, lower IL-6, hs-CRP at 14 days, and low hemorrhagic transformation incidence within 7 days.
Li et al. (2024) (30)	China	RCT	123	60.33 (±9.57) treatment, 60.73 (±7.9) control	75/48	22.72 (2.86) treatment, 22.04 (2.75) control	N/R	N/R	15 mL mixed with 100 mL 0.9% sodium chloride IV, twice daily for 14 days (Edaravone Dexborneol)	Alteplase, 10% 0.9 mg/kg IV bolus + remaining 90% infusion over 1 h without Edaravone or Dexborneol	90	Analyzed the therapeutic role of edaravone dexborneol in AIS and compared its efficacy and side effects with alteplase therapy.	Favorable NIHSS and mRS scores, fewer side effects, improved ADL (Barthel Index), serum indices analyzed (NO, ET-1, MMP-2).
Xu et al.(2019) (14)	China	RCT (phase II)	385	58.13–59.71 (SD: ~8.3–9.5, across groups)	216/124	~24.22–25.44 (varies by group)	~148.91–150.91	~88.5	Low: 12.5 mg, Medium: 37.5 mg, High: 62.5 mg IV infusion every 12 h for 14 days (Edaravone Dexborneol)	30 mg IV infusion every 12 h for 14 days, Edaravone without Dexbrompheniramine	90	Compared the safety and efficacy of edaravone dexborneol vs. edaravone in AIS patients.	Improved mRS scores ≤1, lower NIHSS scores, better Barthel Index, cognitive assessment (MoCA), and Stroke Impact Scale scores.
Xu et al. (2024) (29)	China	RCT	93	68.49 (±12.579)	65/28	24.73 (2.3)	141.02 ± 19.551	85.52 ± 13.1	15 mL (Edaravone 30 mg + borneol 7.5 mg) IV infusion, twice daily for 14 days	Standardized ischemic stroke treatment without Edaravone or Dexborneol	30	Observed the effect of edaravone dexborneol on early PSD and its inflammatory mechanisms.	Lower PSD incidence, PHQ-9, HAMD scores, lower inflammatory factors at day 14, and improved NIHSS scores at days 14 and 30.
Xu et al. (2021) (6)	China	RCT (phase III)	1,200	61.82 males, 64.88 females	811/383	24.4 + 3.2 treatment, 24.2 + 2.8 control	INT SP = 149 + 24. PL SP = 146 + 24	. INT DP = 88 + 13/PL DP = 86 + 11	14-day infusion (Edaravone Dexborneol)	14-day infusion Edaravone without Dexborneo	90	Tested edaravone dexborneol vs. edaravone on 90-day functional outcomes in AIS patients.	Improved mRS scores ≤1, reduced NIHSS scores, higher Barthel Index scores, fewer adverse events, and better safety profile.

ADL, Activities of Daily Living; BI, Barthel Index; CI, Confidence Interval; DP, Diastolic Pressure; ET-1, Endothelin-1; HAMD, Hamilton Depression Rating Scale; hs-CRP, High-Sensitivity C-Reactive Protein; IL-6, Interleukin-6; IQR, Interquartile Range; Int, Intervention group; mRS, Modified Rankin Scale; MoCA, Montreal Cognitive Assessment; NIHSS, National Institutes of Health Stroke Scale; NO, Nitric Oxide; N/R, Not Reported; PHQ-9, Patient Health Questionnaire-9; PL, Placebo group; PSD, Post-Stroke Depression; RCT, Randomized Controlled Trial; SD, Standard Deviation; SP, Systolic Pressure.

### 3.3 Risk of bias assessment

**Figure 3:**
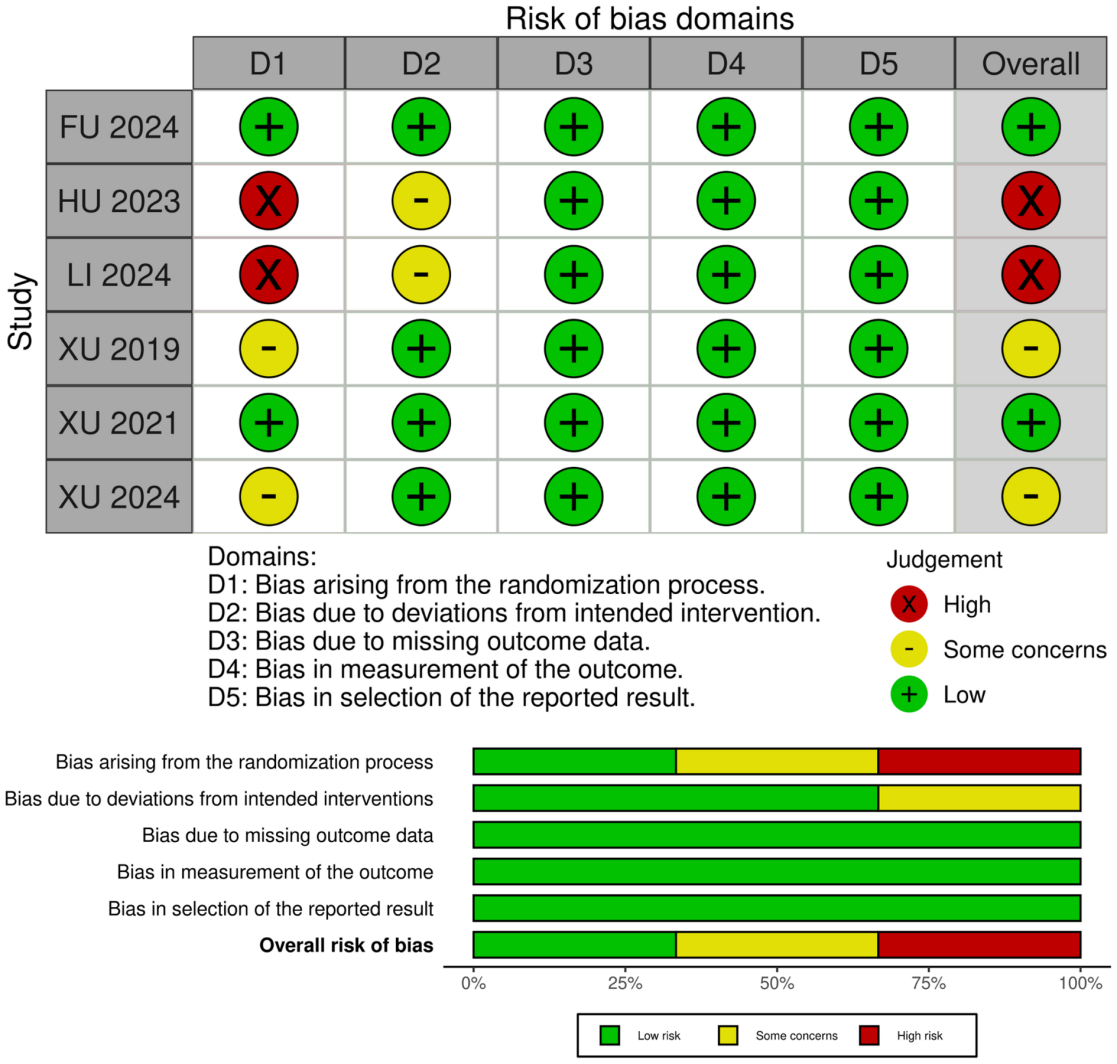
‘Risk of bias graph’: review authors’ judgments about each risk of bias item presented as percentages across all included studies, and ‘Risk of bias summary’: review authors’ judgments about each risk of bias item for each included study.

After the quality evaluation of the seven included studies, there were two low-risk studies (6, 7), two with some concerns (14, 29), and two high-risk studies (30, 31). The quality evaluation form according to the RoB2 scale (the risk of bias was classified into three levels: “low risk of bias, “some concerns,” and “high risk of bias”), as shown in Figure 3. The observational study by Chen et al. (2024) (32) was of good quality, up to the NOS score (9/9).

### 3.4 Outcomes analyzed

#### 3.4.1 National Institutes of Health Stroke Scale

**Figure 4:**
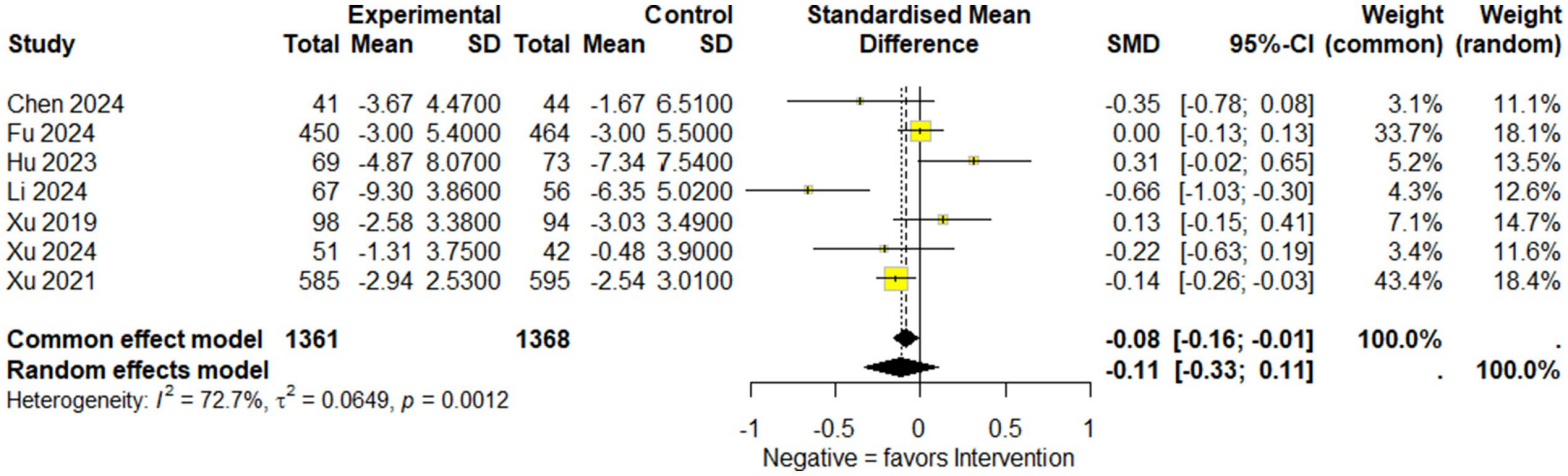
Forest plot showing National Institutes of Health Stroke Scale (NIHSS) available data of included studies.

**Figure 5:**
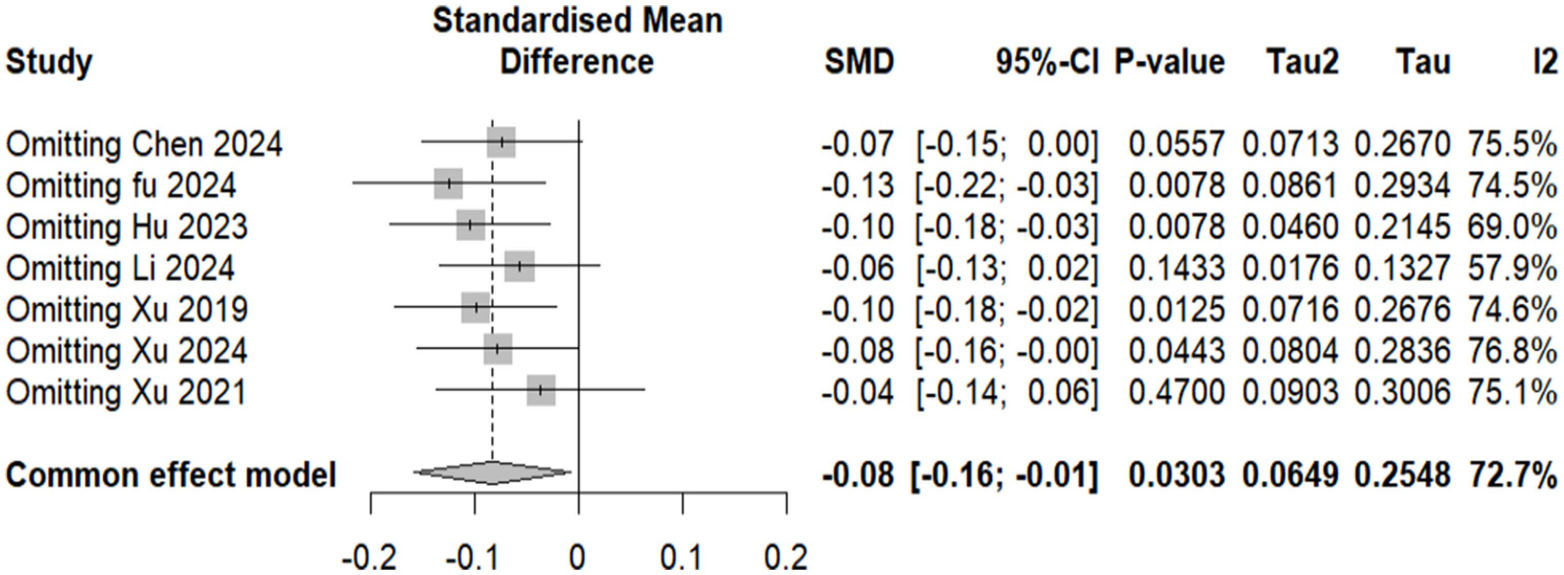
Forest plot showing sensitivity analysis for the National Institutes of Health Stroke Scale (NIHSS) effect size by excluding each study and showing *p*-value.

**Figure 6:**
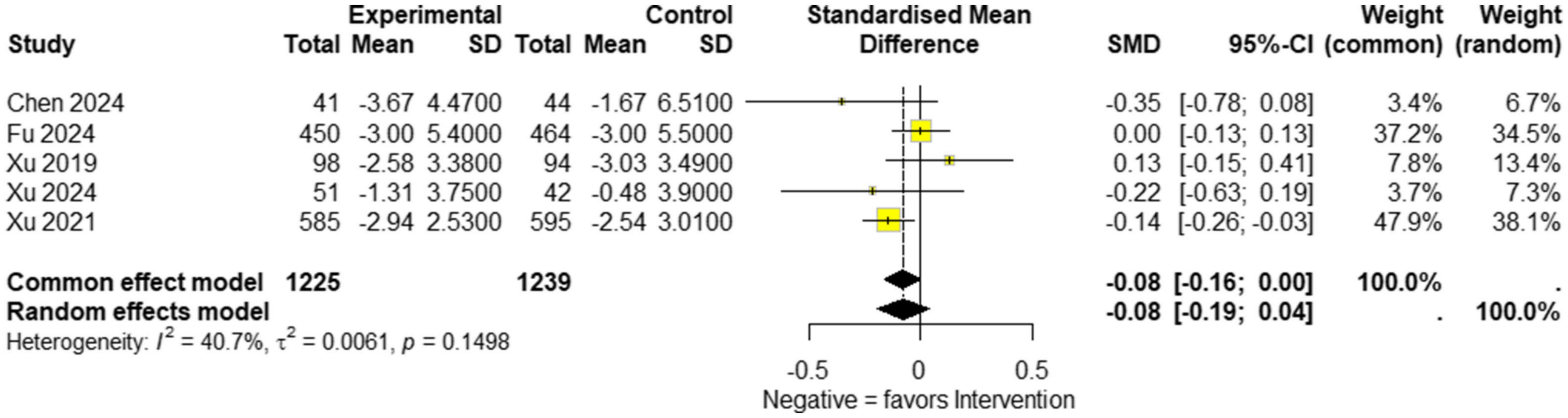
Forest plot showing the National Institutes of Health Stroke Scale NIHSS after excluding Li et al. (5, 30) and Hu et al. (31).

Overall results: A total of seven studies comprising 2,729 participants (1,361 in the experimental group and 1,368 in the control group) were included in the NIHSS analysis. Under the common-effect model, the pooled effect size demonstrated a small but statistically significant improvement in NIHSS scores favoring the intervention (SMD = −0.083, 95% CI: −0.159 to −0.008, *p* = 0.030). However, the random-effects model, which is more reliable, produced a non-significant result (SMD = −0.113, 95% CI: −0.333 to 0.107, *p* = 0.314). Substantial heterogeneity was detected (I^2^ = 72.7%, Q = 22.0, *p* = 0.0012), indicating considerable variability across the included studies. Sensitivity analysis: Leave-one-out sensitivity analysis was conducted to explore the influence of individual studies. The omission of Fu 2024, Hu 2023, Xu 2019, or Xu 2024 strengthened the significance of the pooled estimate, producing clearer evidence of NIHSS reduction (all *p* < 0.05). In contrast, omission of Xu 2021 attenuated the association to non-significance (*p* = 0.47), while exclusion of Li 2024 also weakened statistical significance (*p* = 0.14). These findings suggest that although no single study completely drove the overall effect, certain trials either diluted or reinforced the pooled estimate. Reduced model (excluding Li 2024 and Hu 2023): To further address heterogeneity, a secondary analysis was performed after excluding Li 2024 and Hu 2023, which were identified as major contributors to variability. In this reduced model of five studies (*n* = 2,464), the common-effect model yielded a borderline significant result (SMD = −0.079, 95% CI: −0.158 to 0.0004, *p* = 0.051), while the random-effects model remained non-significant (SMD = −0.077, 95% CI: −0.195 to 0.042, *p* = 0.204). Importantly, heterogeneity decreased from 72.7 to 40.7%, suggesting that exclusion of these two studies improved consistency across trials, though at the cost of reduced statistical significance (Figures 4–6).

#### 3.4.2 Modified Rankin Scale (mRS)

**Figure 7:**
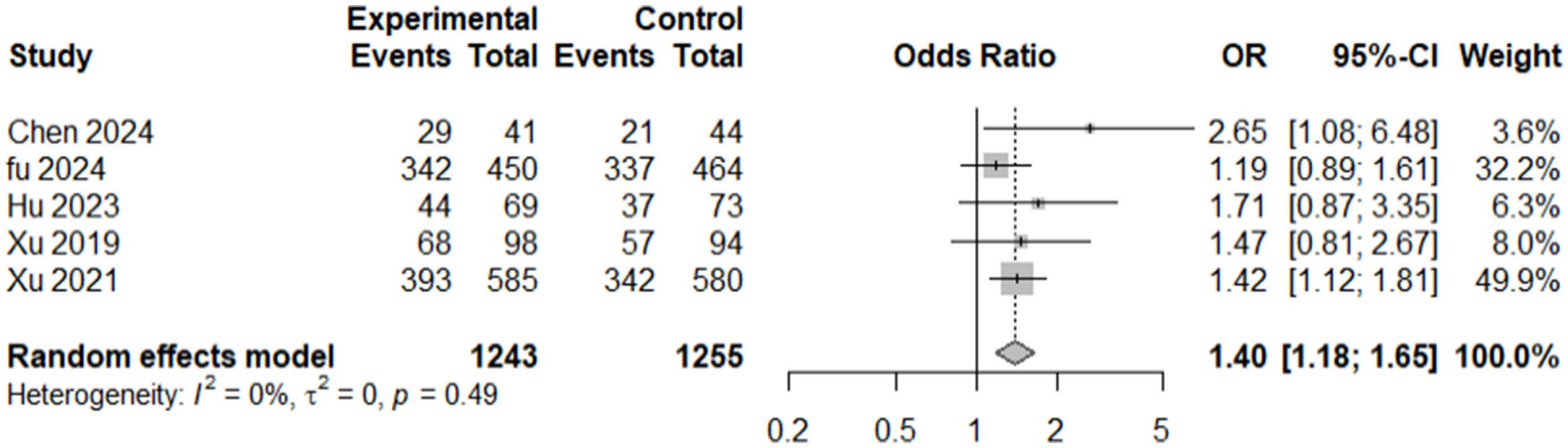
Forest plot showing modified Rankin Scale score (mRS) available data of included studies.

A total of five studies (6, 7, 14, 29–32) reported (mRS) score of ≤ 2 at 90 days, comprising 2,498 participants (1,243 in the intervention group and 1,255 in the control group), revealed that the intervention group had significantly better outcomes than the control group. The random-effects model calculated an odds ratio (OR) of 1.3951 (95% confidence interval [CI]: 1.1783–1.6517, *p* = 0.0001), suggesting 39.5% higher odds of achieving good functional outcomes in the intervention group than in the control. The heterogeneity analysis indicated no substantial variation among the included studies, with τ^2^ = 0 and I^2^ = 0.0%, indicating consistency across studies. The test for heterogeneity (Q = 3.44, *p* = 0.4871) further supported this finding. These results demonstrated a statistically robust and homogenous improvement in functional outcomes with the intervention at 90 days post-treatment. This evidence underscores the potential benefit of the intervention in achieving better recovery, as measured by the mRS score (Figure 7).

### 3.5 Adverse events

Investigations into the use of edaravone dexborneol for acute ischemic stroke (AIS) consistently revealed a favorable safety profile, characterized by mild adverse effects and a low incidence of severe complications (Table 3). Fu (2024) (7) reported similar rates of adverse events between the sublingual edaravone dexborneol group (89.8%) and the placebo group (90.1%), indicating a comparable safety margin. Li (2024) (30) noted that common side effects, including nausea, vomiting, dizziness, chest tightness, fever, and headache, occurred at comparable rates in both the edaravone dexborneol injection and control groups, further underscoring its tolerability. Xu (2019) (14) identified 29 serious adverse events, such as pruritus, skin rash, acute liver injury, and kidney damage, with two severe cases associated with high-dose edaravone dexborneol; however, no significant differences in serious adverse events were observed among the treatment groups. Hu (2023) (31) demonstrated a safety advantage of edaravone dexborneol by significantly reducing the incidence of hemorrhagic transformation (20.29% vs. 39.73% in the control group), a serious complication of AIS. Similarly, Xu (2024) (29) reported only mild adverse effects, such as dizziness and nausea, with no serious reactions noted, whereas Xu et al. (2021) (6) found similar rates of adverse events between the edaravone dexborneol and edaravone groups (54 vs. 47 patients with serious adverse effects, respectively). Collectively, these findings reinforce the consistent safety outcomes associated with edaravone dexborneol, with adverse effects typically being mild, dose-dependent, and on par with those of standard treatments, highlighting its acceptability in the management of AIS.

**Table 3:** Aim and adverse event of studies.

Author, year	Adverse events	Efficacy
Chen et al. (2024) (32)	Adverse events not reported.	A significantly higher proportion of patients in the edaravone dexborneol (EDB) group achieved mRS ≤ 2 at 90 days (70.7% vs. 47.8%, *p* = 0.031). NIHSS scores were significantly lower on days 10–14 (*p* = 0.019).
Fu et al. (2024) (7)	Comparable rates of adverse events: EDB group 89.8% vs. placebo group 90.1%.	EDB group demonstrated improved functional outcomes with mRS ≤ 1 at 90 days (64.4% vs. 54.7%; OR: 1.50, *p* = 0.003). Secondary outcomes indicated a better median mRS for the EDB group (OR: 1.33, *p* = 0.02).
Hu et al. (2023) (31)	Hemorrhagic transformation incidence was significantly lower in the EDB group (20.29%) compared to controls (39.73%).	EDB significantly improved NIHSS and mRS scores, reflecting better neurological function and reduced disability. A higher percentage of patients in the EDB group achieved mRS ≤ 1 at 90 days.
Li et al. (2024) (30)	Reported adverse events included nausea, vomiting, chest tightness, fever, dizziness, and headache. Safety profiles were comparable across groups.	EDB demonstrated superior outcomes with significant reductions in NIHSS and mRS scores compared to alteplase treatment (P < 0.05). Effective rate: EDB 92.54% vs. control 78.57%.
Xu et al. (2019) (14)	Serious adverse events (SAEs) included pruritus, rash, acute liver injury, and kidney injury. Two SAEs related to EDB occurred in one high-dose patient.	No statistically significant differences were observed in mRS ≤ 1 at 90 days (*p* = 0.4054) or NIHSS score changes (*p* = 0.6799). Medium- and high-dose groups showed numerically better outcomes.
Xu et al. (2024) (29)	Adverse events included dizziness (5.9%) and nausea (2.4%) in the EDB group. No serious adverse reactions were reported.	EDB significantly reduced early post-stroke depression (PSD) incidence on day 14 (13.7% vs. 31.0%) and day 30 (15.7% vs. 40.5%). Improved PHQ-9 and HAMD scores were observed in the EDB group.
Xu et al. (2021) (6)	Adverse event rates were comparable: EDB (54 SAEs) vs. edaravone (47 SAEs).	EDB group showed superior efficacy, with mRS ≤ 1 achieved by 67.18% of patients (OR: 1.42, *p* = 0.004). Per-protocol analysis supported these findings (OR: 1.67, *p* = 0.001). NIHSS improvement (−0.40 points, p = 0.01).

AIS: Acute Ischemic Stroke; HT: Hemorrhagic Transformation; EDB: Edaravone Dexborneol; SAE: Serious Adverse Events; PSD: Post-Stroke Depression.

### 3.6 GRADE assessment

The quality of evidence for the outcomes assessed in this review was evaluated using the GRADE of Recommendations Assessment, Development, and Evaluation framework. Among the included studies, the certainty of the evidence ranged from high to very low. The primary outcome, the National Institutes of Health Stroke Scale (NIHSS), was rated as moderate, and the modified Rankin Scale (mRS) was rated as moderate due to the combination of RCTs and observational studies and the moderate quality of overall bias. Overall, the strength of evidence was downgraded for factors such as the Risk of Bias, Inconsistency, Indirectness, Imprecision, and Publication Bias, highlighting the need for further high-quality studies to confirm these findings.

## 4 Discussion

Stroke remains a leading cause of mortality and a significant contributor to global disability, impacting approximately 13.7 million individuals annually, with a resultant 5.5 million deaths (33). This neurological disorder is characterized by notable impairments in motor function, often resulting in weakness or paralysis (34). The failure of various clinical trials (8–12) aimed at investigating neuroprotective agents underscores the challenges encountered in establishing effective treatments for AIS. Trials such as SAINT I and II, which evaluated the free radical–trapping neuroprotectant NXY-059, demonstrated no significant benefits for AIS treatment within 6 h of stroke onset, as reported by Shuaib et al. (11). Similarly, nerinetide did not yield improvements in functional outcomes following endovascular therapy, and the ALIAS trials indicated that a dosage of 25% albumin (2 g/kg IV) failed to enhance 90-day clinical outcomes while elevating the risks of pulmonary edema and intracerebral hemorrhage (9). Furthermore, magnesium sulfate administered within 2 h post-stroke onset did not result in functional improvements at 90 days, as noted by Saver et al. (8). These findings emphasize the urgent need for more effective neuroprotective agents to mitigate disability and mortality rates in the management of AIS.

The primary outcomes analyzed, including the National Institutes of Health Stroke Scale (NIHSS) scores and the modified Rankin Scale (mRS), provide critical evidence regarding the potential benefits of edaravone dexborneol in stroke recovery. The pooled analysis of NIHSS scores across seven studies (*n* = 2,729) showed only a modest treatment effect. A significant benefit was seen under the common-effect model (SMD = −0.083, 95% CI: −0.159 to −0.008, *p* = 0.030), but this was not sustained under random effects (SMD = −0.113, 95% CI: −0.333 to 0.107, *p* = 0.314). High heterogeneity (*I*^2^ = 72.7%) suggests variability in trial designs and patient populations. Sensitivity analyses indicated that certain studies diluted the overall benefit, while a reduced model excluding Li 2024 and Hu 2023 improved consistency (*I*^2^ = 40.7%) but did not yield robust significance. In contrast, the mRS analysis demonstrated consistent and clinically meaningful improvements. Pooled results from five studies showed a 39.5% higher likelihood of achieving favorable functional outcomes (mRS ≤ 2) at 90 days (OR 1.40; 95% CI: 1.18–1.65; *p* = 0.0001), with no heterogeneity (*I*^2^ = 0%). These findings reinforce the reliability of functional recovery benefits despite modest effects on acute neurological scores. Taken together, these results suggest that edaravone dexborneol may not strongly impact short-term neurological deficits but provides more robust improvements in long-term functional independence. This discrepancy likely reflects differences between the NIHSS, which measures acute neurological impairment, and the mRS, which captures overall recovery and daily functioning.

Safety remains a paramount consideration in AIS therapies, and the findings of this review confirm the favorable safety profile of edaravone dexborneol. Adverse events were generally mild and comparable to those associated with standard treatments, as demonstrated by studies conducted by Fu et al. (2024) (7) and Xu et al. (2024) (6, 14, 29). Importantly, the observed reduction in rates of hemorrhagic transformation, as reported by Hu et al. (2023) (31) and Xu et al. (2024) (6, 14, 29), suggests a potential protective effect against severe complications, thereby supporting its acceptability in clinical practice. However, occasional reports of serious adverse events such as acute liver injury and renal damage further underscore the need for careful patient selection and monitoring, particularly at elevated dosages (Xu et al., 2019) (14).

When compared with other neuroprotective agents, edaravone dexborneol demonstrates several notable advantages. Compared to edaravone alone, EDB consistently achieves superior functional outcomes and lower NIHSS scores, likely due to its multimodal antioxidant and anti-inflammatory effects. In contrast, citicoline, though well-tolerated and available in oral formulations, has shown mixed results in large trials, with limited impact on functional independence (35, 36). Nerinetide (NA-1) has produced promising findings in specific subgroups, particularly patients not receiving thrombolysis, but its overall efficacy has been inconsistent. Butylphthalide, another agent widely studied in China, included in a review of comparative efficacy of neuroprotective agents, has demonstrated robust improvements across multiple outcomes, though its availability remains limited outside certain regions (35, 36). From a safety standpoint, EDB shows no significant increase in serious adverse events and may even reduce hemorrhagic transformation risk compared to control, providing an advantage over agents with renal or gastrointestinal concerns, such as edaravone alone or minocycline. In terms of feasibility, oral options like citicoline or butylphthalide may be more practical in outpatient settings, whereas EDB requires intravenous administration over 10–14 days, which could restrict its use in some healthcare systems. Taken together, these comparisons suggest that while EDB is a strong candidate within the neuroprotective landscape, its use may be best optimized in inpatient settings or in combination with reperfusion therapies, with further head-to-head trials needed to clarify its position relative to other agents (35–37).

In addition to safety concerns, publication bias represents a potential issue within this review. The Fail-Safe N test indicated that six additional negative studies would be necessary to nullify the observed effect (*p* = 0.013). Other assessments, including the Begg and Mazumdar Rank Correlation (*p* = 0.773) and Egger’s regression (*p* = 0.465), were either inapplicable or yielded non-significant results due to the limited number of studies. These findings underscore the risk of overestimating positive results attributed to selective publication practices. This bias may be exacerbated by the predominance of studies conducted in China, where negative findings are less likely to be disseminated. To address this issue, future meta-analyses should incorporate comprehensive searches of registries and unpublished datasets. Furthermore, the limited number of included trials and their methodological variability impede the generalizability of the findings (Figure 6 and Table 3). The inclusion of two high-risk studies (Xu et al., 2024; Hu et al., 2023) also impacts the robustness of the conclusions (29, 31).

Our review provides a comprehensive synthesis of the existing evidence on edaravone dexborneol, incorporating both its efficacy and safety outcomes. Notably, this study highlights the benefits of edaravone dexborneol in addressing the secondary outcomes often overlooked in AIS management, such as PSD and inflammatory markers. Xu et al. (2019, 2021) (6, 7, 14, 29–32) Xu et al. (2024) (6, 7, 14, 29–32), and Li et al. (2024) (30) reported consistent reductions in markers such as IL-6, hs-CRP, and MMP-2, which are closely linked to stroke pathophysiology and recovery. These findings align with emerging evidence of the neuroprotective and anti-inflammatory properties of edaravone dexborneol, suggesting broader therapeutic applications beyond motor function recovery. Future research should prioritize integrating these markers into standardized outcome assessments to better elucidate their clinical relevance.

Additionally, this review identified consistent cognitive improvements, including better MoCA and Stroke Impact Scale scores, in trials, such as Xu et al. (2019) (14). These findings underscore the potential of edaravone dexborneol to address cognitive deficits that are often overlooked in AIS management. Incorporating cognitive outcomes into future trials could further refine their role as comprehensive therapeutic options.

### 4.1 Limitations and recommendations for future research

Although this review highlights the promise of edaravone dexborneol as a neuroprotective agent, several important limitations must be acknowledged. First, the small number of available trials substantially limits the robustness and statistical power of the pooled findings, making definitive conclusions premature. Second, considerable heterogeneity existed across studies with respect to dosages, administration routes (intravenous versus sublingual), and comparator treatments. These differences may have influenced the observed variability in outcomes, particularly NIHSS scores, and reduced the certainty of the overall effect estimates. Determining the optimal dosage and administration route through standardized protocols should be a priority for future trials.

Another limitation is that most included studies assessed outcomes only up to 90 days post-treatment, leaving uncertainty about the long-term efficacy and safety of edaravone dexborneol. Extended follow-up durations (e.g., 6–12 months) are necessary to better understand its sustained benefits and risks. Additionally, subgroup analyses stratified by stroke severity, comorbidities, and demographic factors are needed to clarify which patient populations are most likely to benefit. The lack of head-to-head comparisons with other neuroprotective or adjunctive therapies also limits the ability to contextualize edaravone dexborneol within the broader stroke management landscape.

Perhaps the most significant limitation is the geographic concentration of all trials in China. This regional restriction raises concerns regarding external validity and generalizability to more diverse populations, health systems, and clinical practices. To strengthen confidence in these findings, large, high-quality, multicenter RCTs across different countries and ethnic groups are urgently required.

Finally, the overall quality of future studies must be improved. Rigorous randomization, proper blinding, and adherence to standardized reporting guidelines are essential to minimize bias and enhance reliability. Until such data are available, the current evidence should be interpreted with caution, recognizing both its promise and its significant limitations.

## 5 Conclusion

This meta-analysis highlights edaravone dexborneol as a promising therapeutic option for AIS, offering significant functional improvements, a favorable safety profile, and potential neuroprotective benefits. However, the limited number of high-quality studies and lack of global validation necessitate further research to fully establish its role in AIS management. By addressing these gaps and accounting for publication bias, future studies can contribute to the development of more effective treatment strategies, ultimately leading to improved outcomes in patients with AIS.

## Data Availability

The original contributions presented in the study are included in the article/supplementary material, further inquiries can be directed to the corresponding author/s.

## Glossary

RCTs: Randomized Controlled Trials
ADL: Activities of daily living
AIS: Acute Ischemic stroke
PRISMA: Preferred
NIHSS: National Institutes of Health Stroke Scale
MoCA: Montreal Cognitive Assessment
mRS: Modified Rankin Scale
OR: Odd Ratio
CI: Confidence Interval
I^2^: Chi-square
rTPA: Recombinant tissue plasminogen activator
SAINT I/II Trial: Stroke-Acute Ischemic-NXY-Treatment I and II
AhR: Aryl hydrocarbon receptor
RoB: Risk of Bias
NOS: Newcastle-Ottawa Scale
IQR: Interquartile range
SD: Standard deviation
MC: Mean Change
PSD: Post-stroke depression
SMD: Standard mean differences
ALIAS Trial: Albumin in Acute Ischemic Stroke
